# Investigating country-specific music preferences and music recommendation algorithms with the LFM-1b dataset

**DOI:** 10.1007/s13735-017-0118-y

**Published:** 2017-02-06

**Authors:** Markus Schedl

**Affiliations:** Department of Computational Perception, Johannes Kepler University, Altenberger Straße 69, 4040 Linz, Austria

**Keywords:** Music information retrieval, Music recommender systems, Dataset, Statistical analysis, Music taste analysis, Experimentation

## Abstract

Recently, the LFM-1b dataset has been proposed to foster research and evaluation in music retrieval and music recommender systems, Schedl (Proceedings of the ACM International Conference on Multimedia Retrieval (ICMR). New York, 2016). It contains more than one billion music listening events created by more than 120,000 users of Last.fm. Each listening event is characterized by artist, album, and track name, and further includes a timestamp. Basic demographic information and a selection of more elaborate listener-specific descriptors are included as well, for anonymized users. In this article, we reveal information about LFM-1b’s acquisition and content and we compare it to existing datasets. We furthermore provide an extensive statistical analysis of the dataset, including basic properties of the item sets, demographic coverage, distribution of listening events (e.g., over artists and users), and aspects related to music preference and consumption behavior (e.g., temporal features and mainstreaminess of listeners). Exploiting country information of users and genre tags of artists, we also create taste profiles for populations and determine similar and dissimilar countries in terms of their populations’ music preferences. Finally, we illustrate the dataset’s usage in a simple artist recommendation task, whose results are intended to serve as baseline against which more elaborate techniques can be assessed.

## Introduction

In the era of social media platforms and excessive creation of user-generated content, it has never been easier to gather and process digital user traces on a large scale, and in turn exploit them to build comprehensive user profiles. Thanks to this abundance of user and usage data, the research fields of music information retrieval (MIR) and music recommender systems, like many others, are currently in the process of a paradigm shift, away from system-centric approaches and models toward listener-centric ones [12]. At the same time, obtaining meaningful, clean, and large-scale data about listeners, music items, and their interaction is time-consuming and laborious. On the one hand, music platforms such as Spotify,^1^ Last.fm,^2^ or Soundcloud^3^ provide convenient APIs that offer access to their databases. As a consequence, a common strategy in academic research on listener-aware MIR is that researchers acquire experimental data themselves using these APIs, which results in non-standardized data collections, in turn hindering reproducibility.

While there exist a few publicly available datasets to alleviate this problem, e.g., the *Million Song Dataset* (MSD) [2], their usage in listener-centric MIR tasks is restricted. In particular, the MSD provides various pieces of information, including audio content descriptors, editorial metadata, vector space representations of lyrics, tags, and song similarity. In contrast, listener- and listening-related information is given only as aggregated playcount data and liked songs. However, listener-specific information is vital to build personalized music retrieval systems. Therefore, one rationale when creating the LFM-1b dataset was to provide detailed information about listeners and listening events. Examples of such pieces of information include aspects of the listeners’ temporal music consumption behavior, the mainstreaminess of their music taste, and their inclination to listen to unknown music.

We identify several key tasks the LFM-1b dataset can be used for. Given the comprehensive listening data, the most obvious one is music recommendation, which the dataset allows to effect on the artist, album, or track level. In particular, the additional information and computational features about listeners (temporal profiles, novelty, and mainstreaminess) enable the creation of personalized and context-aware recommender systems. Another task we contemplate is music retrieval by time or location. We recently presented a user interface dubbed *Music Tweet Map* [5] for this task. In addition, the dataset in its current version can be used to model music taste on the level of user groups (e.g., based on age or gender) or countries, which opens opportunities to analyze variations and evolutions in music preferences and—complemented with publicly available data on cultural or socioeconomic aspects of populations—even to predict these music preferences from such data [15]. These predictions in turn can be used to remedy the cold-start problem in recommender systems. For future versions of the LFM-1b dataset, we plan to incorporate additional information, such as musical descriptors computed from the audio or text features extracted from web pages or from lyrics. Audio descriptors will enable tasks such as music identification or content-based recommender systems. Text-based features will pave the way to semantic querying by lyrics or by artist characteristics.

This article is structured as follows. We first review related datasets for music retrieval and recommendation (Sect. 2). Subsequently, we outline the data acquisition procedure, present the dataset’s structure and content, provide basic statistics and analyze them, and point to sample Python scripts that show how to access the components of the dataset (Sect. 3). Hereafter, we present an investigation of the music taste in different countries of the world, exploiting demographic information in the dataset (Sect. 4). We further illustrate how to exploit the dataset for the use case of building a music recommender system that implements various recommendation algorithms (Sect. 5). Eventually, we conclude the article with a summary and discussion of future research directions (Sect. 6).

The main novel contribution of this article, in comparison to [11], is the detailed analysis of country-specific music genre profiles, provided in Sect. 4. We show how the LFM-1b dataset can be enriched by Last.fm tags and how to model respective music preferences per country using two genre taxonomies. Furthermore, we analyze the resulting genre profiles for selected countries and point to similarities and dissimilarities between countries. We also exploit this information to rank countries according to the mainstreaminess of their populations’ genre preferences.

## Related datasets

The need for user-aware and multimodal approaches to music retrieval and recommendation has been acknowledged many times and is meanwhile widely accepted [8, 10, 16, 17]. However, respective scientific work is still in its fledgling stage. One of the reasons for this is that involving users, which is an obvious necessity to build user-aware approaches, is time-consuming and hardly feasible on a large scale—at least not in academia. As a consequence, datasets offering user-specific information are scarce.

On the other hand, thanks to evaluation campaigns in the fields of music information retrieval and music recommendation, including the *Music Information Retrieval Evaluation eXchange* 4 (MIREX) and the *KDD Cup 2011* 5 [4], the research community has been given several datasets that can be used for a wide range of MIR tasks, from tempo estimation to melody extraction to emotion classification. Most of these datasets, however, are specific to a particular task, e.g., onset detection or genre classification. What is more, for content-based or audio-based approaches, the actual audio can typically not be shared, because of restrictions imposed by intellectual property rights.

Datasets that can be used to some extent for evaluating personalized approaches to music retrieval and recommendation include the *Yahoo! Music* dataset [4], which probably represents the largest currently available music recommendation dataset, containing more than 262 million ratings of more than 620 thousand music items created by more than one million users. The ratings cover a time range from 1999 to 2010. However, the dataset is completely anonymized, i.e., not only users, but also items are unknown. The absence of any descriptive metadata and ignorance of music domain knowledge therefore restricts the usage of the dataset to rating prediction and collaborative filtering [14].

The *Million Song Dataset* 6 (MSD) [2] is perhaps one of the most widely used datasets in MIR research. It offers a wealth of information, among others, audio content descriptors such as tempo, key, or loudness estimates, editorial item metadata, user-generated tags, term vector representations of lyrics, and playcount information. While the MSD provides a great amount of information about one million songs, it has also been criticized, foremost for its lack of audio material, the obscurity of the approaches used to extract content descriptors, and the improvable integration of the different parts of the dataset. The *MSD Challenge* 7 [9] further increased the popularity of the dataset. Organized in 2012, the goal was to predict parts of a user’s listening history, given another part.

Providing more than one million temporally and spatially annotated listening events that have been extracted from microblogs, the *Million Musical Tweets Dataset* 8 (MMTD) [6] particularly supports context-aware recommendation [1]. Each listening event is accompanied by longitude and latitude values, as well as month and weekday. A major shortcoming of this dataset is its uneven geographical distribution of listening events, which is caused by the likewise skewed distribution of microblogging activity around the world.

Another related dataset is constituted of Last.fm data provided by Celma [3]. The dataset comprises two subsets, one containing listening information for about 360 thousand users, only including artists they most frequently listened to. The other subset offers full listening data of nearly a thousand users, where each listening event is annotated with a timestamp, artist, and track name. Both subsets include gender, age, country, and date of registering at Last.fm, as provided by their API.

Other datasets related to a smaller extent to LFM-1b include the *AotM-2011* dataset of playlists extracted from Art of the Mix^9^ as well as the *MagnaTagATune* 10 dataset [7] of user-generated tags and relative similarity judgments between triples of tracks. A more comprehensive discussion of datasets for music recommendation and related tasks can be found in [14].

In comparison to the datasets most similar to the LFM-1b dataset, i.e., the MSD and Celma’s [3], LFM-1b offer the following unique features: (i) substantially more listening events, i.e., over one billion, in comparison to roughly 48 and 19 million, respectively, for MSD and Celma’s [3]; (ii) exact timestamps of each listening event, unlike MSD; (iii) demographic information about listeners in an anonymous way, unlike MSD; and (iv) additional information describing the listeners’ music preferences and consumption behavior, unlike both MSD and Celma’s [3]. These additional descriptors include temporal aspects of listening behavior as well as novelty and mainstreaminess scores as proposed in [13], among others.

**Table 1:** Description of the files constituting the LFM-1b dataset

File	Content
LFM-1b_users.txt	*User-id*, country, age, gender, playcount, registered_timestamp
LFM-1b_users_additional.txt	*User-id*, novelty_artist_avg_month, novelty_artist_avg_6months, novelty_artist_avg_year, mainstreaminess_avg_month, mainstreaminess_avg_6months, mainstreaminess_avg_year, mainstreaminess_global, cnt_listeningevents, cnt_distinct_tracks, cnt_distinct_artists, cnt_listeningevents_per_week, relative_le_per_weekday1, ...relative_le_per_weekday7, relative_le_per_hour0, ...relative_le_per_hour23
LFM-1b_artists.txt	**Artist-id**, artist-name
LFM-1b_albums.txt	***Album-id***, album-name, **artist-id**
LFM-1b_tracks.txt	Track-id, track-name, **artist-id**
LFM-1b_LEs.txt	*User-id*, **artist-id**, ***album-id***, track-id, timestamp
LFM-1b_LEs.mat	Idx_users (vector), idx_artists (vector), LEs (sparse matrix)

Attributes of same emphasis are connected to each other

**Table 2:** Description of the additional user features on preference and consumption behavior

Attribute	Description
user-id	User identifier
novelty_artist_avg_month	Novelty score according to [13], i.e., percentage of new artists listened to, averaged over time windows of 1 month
novelty_artist_avg_6months	Novelty score, averaged over time windows of 6 months
novelty_artist_avg_year	Novelty score, averaged over time windows of 12 months
mainstreaminess_avg_month	Mainstreaminess score according to [13], i.e., overlap between the user’s listening history and an aggregate listening history of all users, averaged over time windows of 1 month
mainstreaminess_avg_6months	Mainstreaminess score, averaged over time windows of 6 months
mainstreaminess_avg_year	Mainstreaminess score, averaged over time windows of 12 months
mainstreaminess_global	Mainstreaminess score, computed for the entire period of the user’s activity on Last.fm
cnt_listeningevents	Total number of the user’s listening events (playcounts) included in the dataset
cnt_distinct_tracks	Number of unique tracks listened to by the user
cnt_distinct_artists	Number of unique artists listened to by the user
cnt_listeningevents_per_week	Average number of listening events per week
relative_le_per_weekday[1–7]	Fraction of listening events for each weekday (starting on Monday) among all weekly plays, averaged over the user’s entire listening history
relative_le_per_hour[0–24]	Fraction of listening events for each hour of the day (starting with the time span 0:00–0:59) among all 24 h, averaged over the user’s entire listening history

## Description of the LFM-1b dataset

In the following, we outline the data acquisition procedure, describe in detail the dataset’s components, analyze basic statistical properties of the dataset, provide download links, and refer to some sample code in Python, which is also available for download. Please note that the LFM-1b dataset is considered derivative work according to paragraph 4.1 of Last.fm’s API Terms of Service.^11^

### Data acquisition

We first use the overall 250 top tags^12^ to gather their top artists^13^ using the Last.fm API. For these artists, we fetch the top fans, which results in 465,000 active users. For a randomly chosen subset of 120,322 users, we then obtain their listening histories.^14^ For approximately 5,000 users, we cap the fetched listening histories at 20,000 listening events in order to avoid ending up with an extraordinarily uneven user distribution (cf. Sect. 3.3), in which a few users have an enormous amount of listening events. We define a listening event as a quintuple specified by user, artist, album, track, and timestamp. The period during which we fetched the data ranges from January 2013 to August 2014.

### Dataset availability and content

The LFM-1b dataset of approximately 8 GB can be downloaded from www.cp.jku.at/datasets/LFM-1b. For ease of access and compatibility, the metadata on artists, albums, tracks, users, and listening events are stored in simple text files, encoded in UTF-8, while the user-artist-playcount matrix is provided as sparse matrix in a Matlab file, which complies to the HDF5 format. This makes the matrix also accessible from a wide range of programming languages. For instance, Python code for data import is provided along with the dataset (cf. Sect. 3.4).

Table 1 gives an overview of the dataset’s content, in particular the included files and respective pieces of information. Keys that are linked to each other are depicted in the same emphasis. Files LFM-1b_artists.txt, LFM-1b_albums. txt, and LFM-1b_tracks.txt contain the metadata for artists, albums, and tracks, respectively. File LFM-1b_LEs. txt contains all listening events, described by user, artist, album, and track identifiers. Each event is further attached a timestamp, which is encoded in Unix time, i.e., seconds since January 1, 1970 (UTC). File LFM-1b_LEs.mat contains the user-artist-playcount matrix (UAM) as Matlab file in HDF5 format. It comprises three items: (i) a 120,175-dimensional vector (idx_users), each element of which links to the user-ids in files LFM- 1b_users.txt, LFM-1b_LEs.txt, and LFM-1b _users_additi onal.txt, (ii) a 585,095- dimensional vector (idx_artists), whose elements link to the artist-ids in LFM-1b_LEs.txt and the metadata files, and (iii) a $120{,}175 \times 585{,}095$ sparse matrix (LEs), whose rows correspond to users and columns to artists. User-specific information is given in LFM-1b_users.txt and LFM-1b_users_additi onal.txt. While the former contains basic demographic information as well as overall playcount and date of registration with Last.fm, the latter provides 43 additional user descriptors that represent a unique feature of LFM-1b. Table 2 describes these user features, which are particularly valuable when creating user-aware music recommender systems.

### Dataset statistics

Table 3 shows basic statistics of the dataset’s composition. The number of unique <user, artist> pairs corresponds to the number of entries in the UAM, which is a $120{,}175 \times 585{,}095$ sparse matrix. Note that these numbers are smaller than the total numbers of unique users and artists reported in Table 3 since we discarded users who listened to less than 10 unique artists and artists listened to by less than 10 users when creating the UAM. We assume that data about these artists and users are too sparse to be informative or contain just noise. In particular, this approach effectively filters artists that are misspelled, which is evidenced by the substantial reduction in their number by $81.66\%$ (from 3,190,371 to 585,095). The reduction in terms of users is much smaller (by $0.21\%$, from 120,322 to 120,175), because users with such a narrow music artist taste are almost nonexistent on Last.fm. This filtering step yields a UAM that is very well manageable with today’s computers (approximately 200 MB).

**Table 3:** Statistics of items in the dataset

Item	Number
Users	120,322
Artists	3,190,371
Albums	15,991,038
Tracks	32,291,134
Listening events	1,088,161,692
Unique <user, artist> pairs	61,534,450

**Table 4:** Statistics on country distribution of users. All countries with more than 1000 users are shown

Country	No. of users	Pct. in dataset (%)
US	10,255	18.581
RU	5024	9.103
DE	4578	8.295
UK	4534	8.215
PL	4408	7.987
BR	3886	7.041
FI	1409	2.553
NL	1375	2.491
ES	1243	2.252
SE	1231	2.230
UA	1143	2.071
CA	1077	1.951
FR	1055	1.912
N/A	65,132	54.131

In the following, we present a more detailed analysis of the demographic coverage, distribution of listening events, and features related to music preference and consumption behavior.

#### Demographics

We compute and illustrate the distribution of users among country, age, and gender. Table 4 shows the countries where most users in the dataset originate from. We include all countries with more than 1,000 users. As can be seen, a majority of users do not provide country information ($54.13\%$). The country-specific percentages in the last column of the table are computed only among those users who provide their country. The distribution of users in the dataset reflects that of Last.fm users in general.

**Fig. 1:**
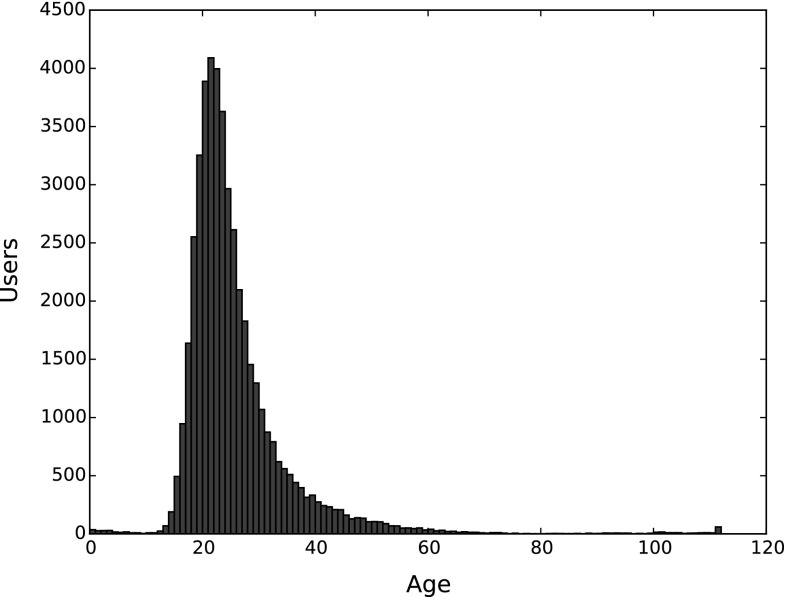
Histogram of age distribution

A histogram illustrating the age distribution is given in Fig. 1. Among all users, only $38.31\%$ provide this piece of information. It can be seen that the age distribution is quite uneven and skewed toward the right (higher ages), but reflects the composition of Last.fm users. In addition to this, we can spot some seemingly erroneous information provided by some users, i.e., 165 of them indicated an age smaller or equal to 6 years, 149 indicated an age of at least 100 years. However, the share of these users only represents $0.26\%$ of all users in the dataset. The age distribution has its arithmetic mean at 25.4 years, standard deviation of 9.7, a median of 23, and 25- and 75-percentile, respectively, at 20 and 28 years.

Table 5 depicts the gender distribution of users in the dataset. Among those who provide this information, more than two thirds are male, less than one third female. The larger share of male users on Last.fm is a known fact. The number of users who provide information on their gender (64,551 or 53.6%) is very close to the number of users who provide country information (65,132 or 54.1%), and considerably higher than the amount of users who indicate their age (46,095 or 38.3%). Therefore, users seem to be highly reluctant to reveal their age.

**Table 5:** Statistics on gender distribution of users

Gender	No. of users	Pct. in dataset (%)
Male	39,969	71.666
Female	15,802	28.334
N/A	64,551	53.649

#### Listening events

**Fig. 2:**
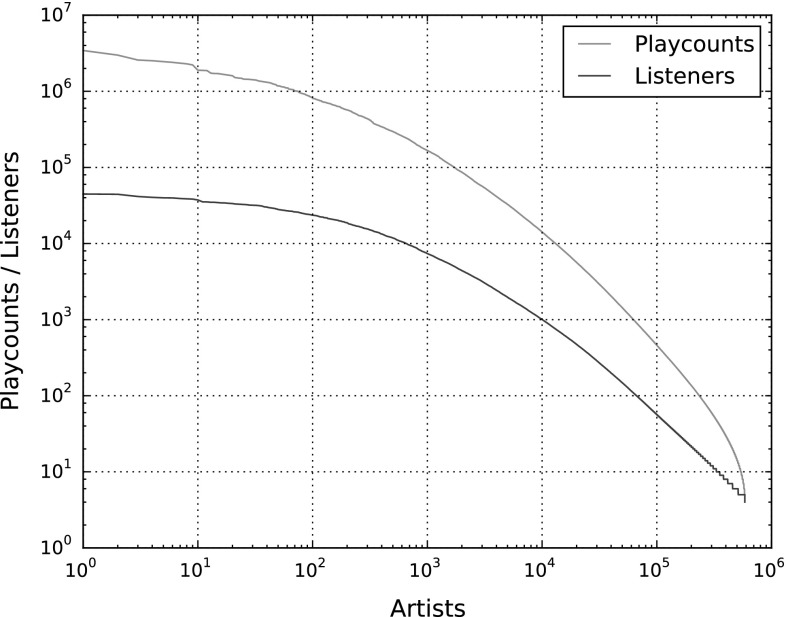
Distribution of listening events by artist, log–log-scaled

**Fig. 3:**
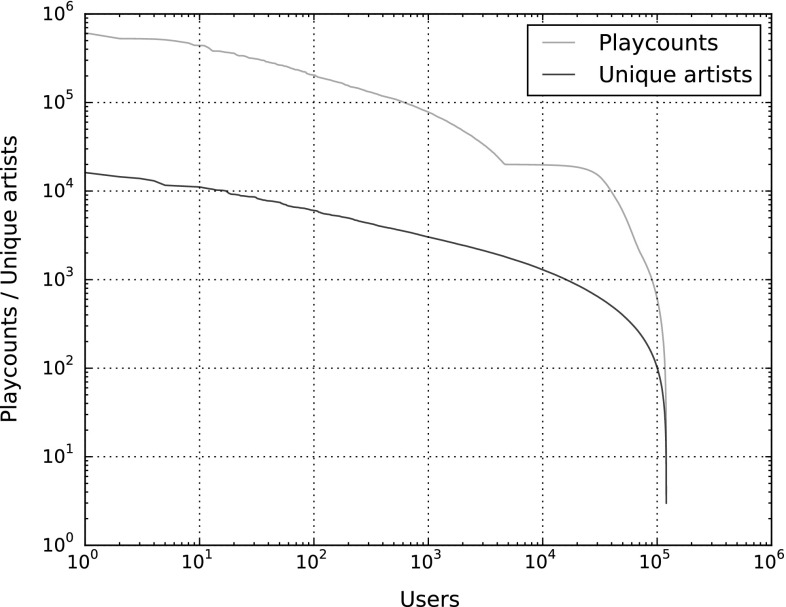
Distribution of listening events by user, log–log-scaled

To gain an understanding of the distribution of listening events in the dataset, Figs. 2 and 3 illustrate the sorted amount of listening events for all artists and for all users, respectively, plotted as red lines. The blue plots indicate the number of listeners each artist has (Fig. 2) and the number of artists each user listens to (Fig. 3). The axes in both figures are logarithmically scaled.

From Fig. 2, we observe that especially in the range of artists with extraordinarily high playcounts (left side of the figure), the number of playcounts decreases considerably faster than the number of listeners. For instance, the top-played artist is on average listened to 78.92 times per user, while the 1,000th most popular artist is listened to only 22.66 times per user, on average. On the other side, the 100,000 least popular artists are played only 1.99 times on average. This provides strong evidence of the “long tail” of artists [3].

From Fig. 3, we see that highly active listeners (in the left half of the figure) tend to have a rather stable relationship between total playcounts and number of artists listened to, whereas the average number of playcounts per artist strongly decreases for less active listeners. Indeed, the 1,000 most active listeners aggregate on average 29.73 listening events per artist, while for the 1,000 least active listeners, this number is only 3.04. Therefore, highly active users tend to listen to tracks by the same artists over and over again, while occasional and seldom listeners tend to play only a few tracks by their preferred artists. Furthermore, we can observe in Fig. 3 the considerable number of users for which we recorded approximately 20,000 listening events, for the reasons given in Sect. 3.1.

Table 6 shows additional statistics of the listening event distribution, both from a user and an artist perspective (second and third column, respectively). The first row shows the average number and standard deviation of playcounts, per user and per artist, computed from the values of the red plots in Figs. 2 and 3. The second row shows the average number of unique artists per user (second column) and the average number of unique users per artist (third column). These numbers are computed from the blue lines in the figures. The third row reveals how often, on average, users play artists they listen to (second column) and how often artists are listened to by users who listen to them at all, on average (third column). The last row is similar to the third one, but uses the median instead of the arithmetic mean to aggregate average playcounts. It shows that there exist strong outliers in the average playcount values, both per user and per artist, because the median values are much smaller than the mean values. For instance, users listen to each of their artists on average about 21 times, but half of all users listen to each of their artists on average only five times or less. Therefore, there are a few users who keep on listening to their artists over and over again, while a large majority do not listen to the same artist more than a few times, on average.

**Table 6:** Statistics of the distribution of listening events among users and artists

	Users	Artists
Playcount (PC)	$8879 \pm 15{,}962$	$1824 \pm 24{,}745$
Unique artists/users	$512 \pm 622$	$105 \pm 733$
Mean PC per artist/user	$21.21 \pm 46.68$	$7.89 \pm 17.83$
Median PC per artist/user	$5.16 \pm 19.35$	$2.50 \pm 2.98$

Values after the ± sign indicate standard deviations

#### Descriptors of preference and consumption behavior

**Fig. 4:**
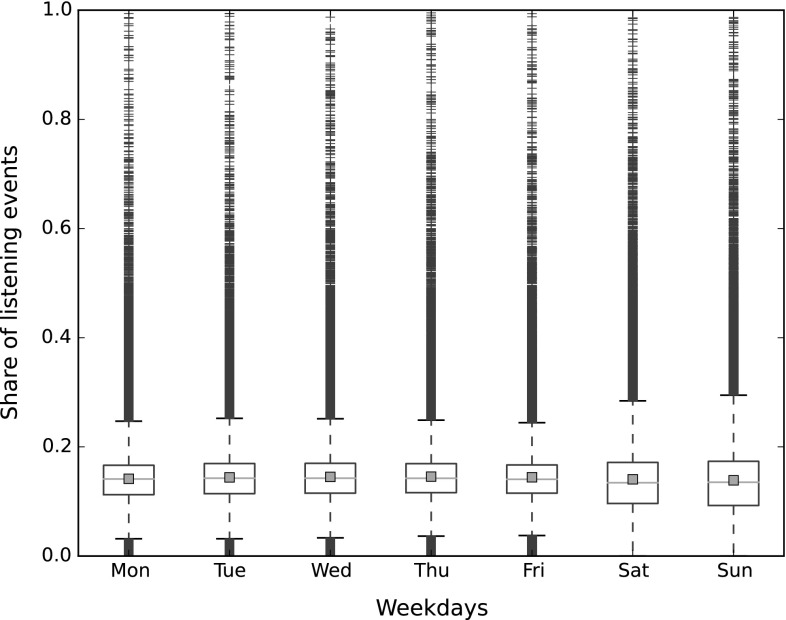
Distribution of listening events over weekdays

**Fig. 5:**
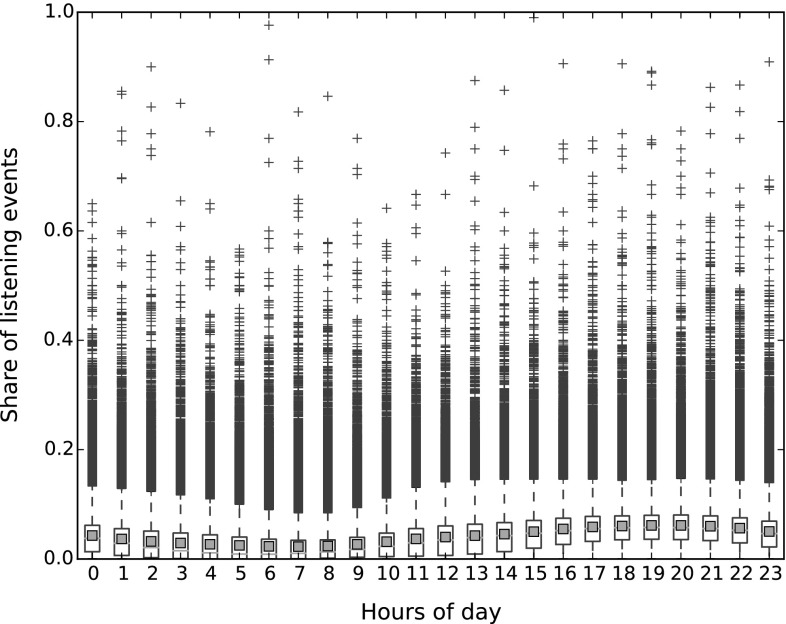
Distribution of listening events over hours of day. Each time range encompasses 0–59 min after the hour indicated on the *x*-axis

The LFM-1b dataset provides a number of additional user-specific features (cf. Table 2), in particular information about temporal listening habits and music preference in terms of mainstreaminess and novelty [13]. To characterize temporal aspects, we binned the listening events of each user into weekdays and into hours of the day, and computed the share of each user’s listening events over the bins. The distribution of these shares is illustrated in Fig. 4 for weekdays and in Fig. 5 for hours of the day. These box plots illustrate the median of the data by a horizontal red line. The lower and upper horizontal black lines of the box indicate the 25- and 75-percentiles, respectively. The horizontal black lines further above or below represent the furthest points not considered outliers, i.e., points within 1.5 times the interquartile range. Points beyond this range are depicted as blue plus signs. The red squares illustrate the arithmetic mean.

We can observe in Fig. 4 that the share of listening events does not substantially differ between working days. However, during weekend (Saturday and Sunday), there is a much larger spread. A majority of people listens less during weekends than during working days (lower median). At the same time, the top $25\%$ of active listeners consume much more music during weekends (higher 75-percentile for Saturday, and even higher for Sunday). This is obviously the result of working and leisure habits.

In Fig. 5, we see that the distribution of listening events over hours of day vary more than over weekdays. It is particularly low during early morning hours (between 4 and 7 h) and peaks in the afternoon and early evening (between 17 and 22 h) when many people indulge in leisure time activities.

To compute the listener scores for novelty and mainstreaminess, we follow the approach presented in [13]. For *novelty*, we split user *u*’s listening history into time windows of fixed length and calculate the percentage of new items listened to, i.e., items appearing for the first time in *u*’s listening history. The novelty $N_{ut}$ of *u*’s listening events in time window *t* is defined as $N_{ut} = \frac{|\{l \in L_{ut} \; \wedge \; l \notin L_{ux} \forall x<t \}|}{| L_{ut} |}$, where $L_{ut}$ is the entirety of items *u* listened to in time window *t*, including duplicates, and $l \notin L_{ux} \forall x<t$ denotes all listening events not listened to by *u* at any time before *t*. Averaging over all time windows user *u* was active in, we obtain *u*’s overall novelty score $N_{u}$. In the LFM-1b dataset, we provide novelty scores for time windows of 1, 6, and 12 months. To quantify the *mainstreaminess* $M_{ut}$ of a user *u* in time window *t*, we relate *u*’s distribution of playcounts over artists to the global playcount distribution of all users: $M_{ut} = \sum _{a \in A}{\sqrt{ \frac{p_{uat}}{p_{ut}} \cdot \frac{p_{at}}{p_{t}}} }$, where $p_{uat}$ is the frequency user *u* listens to each artist *a* in the global playcount vector *A* in time window *t*, $p_{ut}$ and $p_{at}$ represent the total number of playcounts of user *u* and artist *a* in time window *t*, respectively, and $p_t$ denotes the sum of all playcounts in *t*. We again average over all time windows to compute an aggregate mainstreaminess score $M_u$ for user *u*. The scores in the LFM-1b set are provided for time windows of 1, 6, and 12 months, as well as on a global scale. The main statistics of the novelty and the mainstreaminess scores (both computed on time windows of 12 months) are given in Table 7. We can see that most users are eager to listen to new music since the average share of new artists listened to every year is approximately $50\%$. On the other hand, their music taste tends to be quite diverse and far away from the mainstream since the average overlap between the user’s distribution of listening events and the global distribution is only $5\%$.

**Table 7:** Statistics of novelty and mainstreaminess scores

	Novelty	Mainstreaminess
Min.	0.000	0.000
25-perc.	0.354	0.016
Median	0.496	0.045
75-perc.	0.647	0.079
Max.	1.000	0.393
Mean	0.504	0.054
Std.	0.211	0.048

### Sample source code

To facilitate access to the dataset, we provide Python scripts that show how to load the data and perform simple computations, e.g., basic statistics, as well as how to implement a basic collaborative filtering music recommender. The code package can be found on http://www.cp.jku.at/datasets/LFM-1b. File LFM-1b_stats.py shows how to load the UAM, compute some of the statistics reported in Sect. 3.3, and store them in a text file. Based on this text file, LFM-1b_plot.py demonstrates how to create plots such as the one shown in Figs. 2 and 3. In addition, we implement a simple memory-based collaborative filtering approach in LFM-1b_recommend-CF.py, which might serve as reference implementation and starting point for experimentation with various recommendation models.

## Analysis of country-specific music preferences

The demographic information about listeners’ nationalities enables further investigations concerning the music taste of populations. For this purpose, we create country-specific genre profiles. First, the top tags assigned to each artist in the LFM-1b dataset are fetched via the respective Last.fm API endpoint.^15^ These tags provide different pieces of information, including instruments (“guitar”), epochs (“80s”), places (“Chicago”), languages (“Swedish”), and personal opinions (“seen live” or “my favorite”). To gauge music taste, we focus on tags that encode genre and style information and use these descriptors as proxy to model genre profiles per country. To this end, we use two dictionaries, one of 20 general genres used by Allmusic^16^ and one of 1,998 genre and style terms retrieved from Freebase.^17^ We subsequently index the Last.fm artist tags using these two dictionaries separately. Table 8 shows the countries with the highest absolute number of distinct genre and style terms, as well as the relative figures, when indexing with the Freebase dictionary.^18^ We see that the genre coverage is quite high, in particular considering that the Freebase dictionary contains a lot of very specific genres and styles (e.g., “progressive psytrance”, “technical death metal”, or “Ramkbach”).

**Table 8:** Number of distinct genres and styles used by populations of different countries (absolute and relative to the 1998 genres in the Freebase list)

Country	Genres (abs.)	Genres (rel.) (%)
US	1111	55.55
UK	1103	55.15
DE	1100	55.00
RU	1097	54.85
NL	1081	54.05
PL	1077	53.85
SE	1062	53.10
BR	1053	52.65
ES	1043	52.15
FI	1042	52.10

The genre profiles of countries directly relate to the musical preferences of their citizens. Defining a genre listening event as a listening event whose involved artist is tagged with the respective genre, we compute the share of each genre’s listening events among all listening events in a given country. Using either the Allmusic or the Freebase genre list, the resulting genre profile, i.e., distribution of listening events over genres, can be regarded as a coarse or fine-grained description of the population’s music taste, respectively.

### Coarse genre profiles

**Fig. 6:**
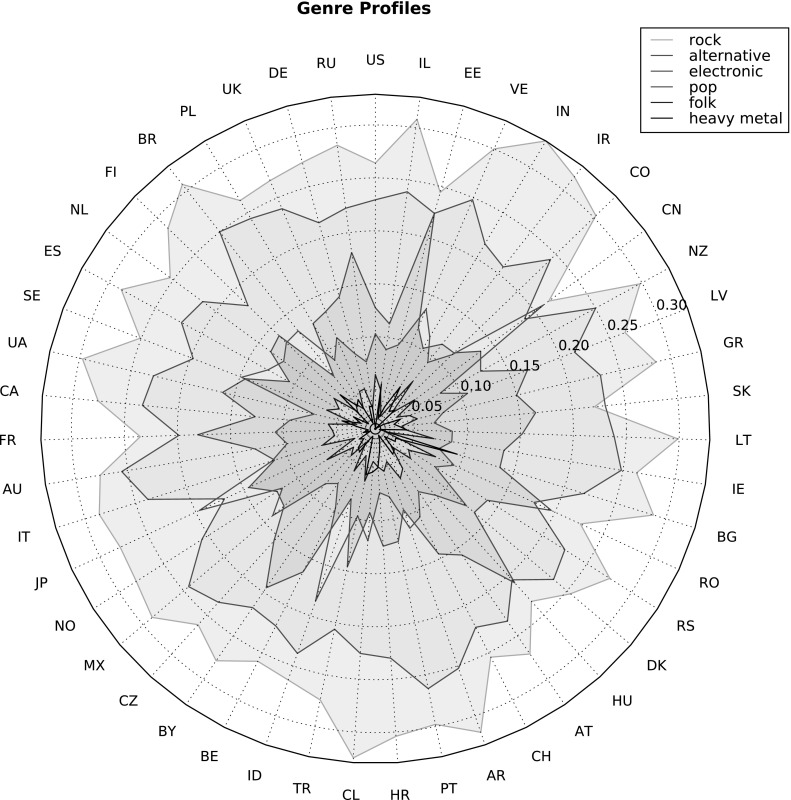
Radar plot of genre profiles for the top 47 countries and most important genres in the Allmusic dictionary

Figure 6 shows a radar plot of the genre profiles according to the Allmusic dictionary, for the 47 countries with at least 100 users in the LFM-1b dataset. Starting with the USA, countries are sorted in descending order of users in a counterclockwise manner. To reduce visual clutter and increase readability, we include only the shares of some of the most popular genres. As a general tendency, we observe that the popularity ranking of genres is quite consistent between countries. A few exceptions are, for instance, Japan and China, where the share of pop music is higher than that of alternative. Interestingly, in the case of China, this larger amount of pop music comes at the expense of rock and alternative, whereas in Japan, only alternative, but not rock, seems to be negatively affected. Electronic music is consumed to a disproportionately high amount in Russia, France, Belarus, Hungary, Romania, and Estonia, whereas very little in South American countries (Brazil, Chile, and Argentina), Indonesia, and India. Pop music peaks in Japan, China, and Indonesia; folk in the USA, Romania, Ireland, and Iran. Metal is particularly popular in Finland, Turkey, and Bulgaria.

### Fine-grained genre profiles

To dig a bit deeper into the music tastes of populations, we create fine-grained genre profiles using the Freebase dictionary, as described above. Table 9 shows the top genres for selected countries. Please note that the reported shares in percentage, scaled to the range [0, 1], are much lower than those in Fig. 6, because the distributions are computed over many more genres and styles. While the very top ranks are, not very surprisingly, occupied by rather broad genres, some interesting observations can be made. In particular, a few rather specific genres, such as UK 82, J-pop, Chill out, or Ambient occur among the top 10 in the United Kingdom, Japan, China, and Iran, respectively. However, one has to bear in mind that the data in the LFM-1b dataset in general, and in particular for countries with restricted access to certain online services, are likely not representative for the respective population at large.

**Table 9:** Relative amount of listening events of the ten most frequent genres and styles for selected countries, using the Freebase dictionary

UK		Japan		China		Iran	
Genre tag	LEs	Genre tag	LEs	Genre tag	LEs	Genre tag	LEs
Rock	0.037763	Rock	0.037155	Rock	0.036785	Rock	0.042020
Indie	0.028798	Alternative	0.033602	Alternative	0.033730	Alternative	0.037685
Pop	0.028575	Pop	0.031633	Pop	0.032775	Metal	0.029204
Alternative rock	0.025095	J-pop	0.028724	Electronic	0.026281	Experimental	0.026783
Electronic	0.022812	Indie	0.025772	Indie	0.025942	Alternative rock	0.023297
Indie rock	0.022592	Electronic	0.023923	Singer-songwriter	0.021522	Indie	0.021951
Experimental	0.020482	Alternative rock	0.020440	Pop rock	0.018610	Progressive	0.021625
Singer-songwriter	0.017092	Experimental	0.018628	Alternative rock	0.018543	Ambient	0.020136
Electronica	0.016494	Electronica	0.018051	Chill out	0.018081	Electronic	0.019818
UK 82	0.016274	Pop rock	0.016519	Experimental	0.016750	Pop	0.019022

### Country similarity according to music preferences

**Fig. 7:**
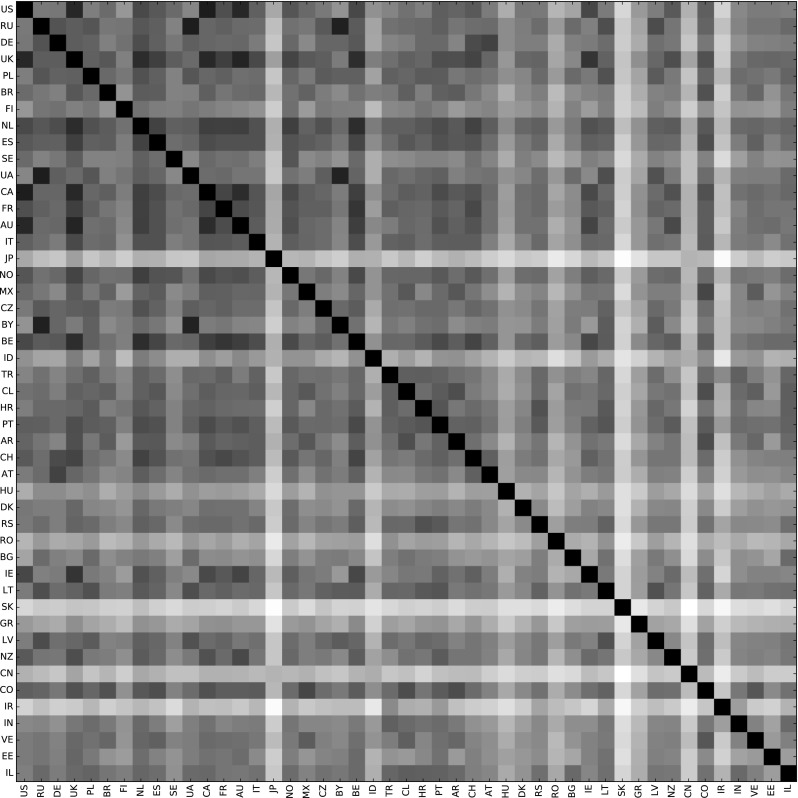
Similarities between selected countries according to Freebase genre profiles

To investigate how similar or dissimilar the music taste of certain populations are, we calculate the cosine similarity between the respective distributions of listening events over genres for all pairs of countries, using the Freebase dictionary. To facilitate the interpretation of results, Fig. 7 encodes these similarities as different shades of gray, where black represents highest and white represents lowest similarity. We can see that the taste in some countries seems to be quite alike. For instance, listeners in the USA, UK, Canada, and Australia tend to share certain genre preferences. So do Russians, Ukrainians, and Belorussians. On the other hand, the figure also reveals countries with a music taste that is highly different from that of most other countries. For example, Japan, Indonesia, Slovakia, China, and Iran show such a characteristic.

By computing, for each country, the arithmetic mean of similarities to all other countries, we can estimate to some extent the mainstreaminess of a population’s music taste. The higher this average country similarity, the closer to many other country’s tastes. Table 10 shows the countries with highest (the Netherlands, UK, Belgium, and Canada) and lowest (Slovakia, Iran, China, and Japan) mainstreaminess among the 47 countries with at least 100 listeners in the dataset.

## Experiments with algorithms for music recommendation

Music recommendation has lately become an important task. While the LFM-1b dataset is not restricted to this task, we illustrate its use for building and evaluating a music recommender system that recommends artists. The following results are intended to serve as baseline for further experimentation and investigating more sophisticated approaches.

### Recommendation algorithms

We implemented several recommendation algorithms, detailed in the following. The results of the experiments are then presented and discussed in Sect. 5.2.

#### Collaborative filtering

A standard memory-based collaborative filtering approach that computes the inner product of the normalized UAM (excluding the artists used for testing) was implemented. After that, the *K* most similar users to the target user, i.e., the user to whom we want to recommend artists, are determined and the artists these *K* neighbors, but not the target user, listened to are weighted with respect to their frequency among the neighbors and the similarity of each neighbor to the target. This process yields a score for each artist which is used to rank them. Finally, the top *N* artists are recommended. For our experiments, we set $K=25$.

#### Demographic filtering

Based on users’ gender, age, and country, we define a user–user similarity matrix, from which we identify the *K* most similar users to the target user and eventually recommend artists using the same weighting as in the CF approach. Demographic similarity is defined binary for gender (1 if same gender, 0 otherwise), and graded for age and country (e.g., 0.8 if the age difference is between 1 and 2 years, 0.2 if the age difference is between 9 and 15 years; 1 if the users reside in the same country, 0.1 if the distance between countries—measured between their midpoint of landmass—is larger than 3,500 km). We then combine these three similarity functions linearly, giving equal weights to all components. Aggregation and recommendation is performed as in the collaborative filtering approach.

**Table 10:** Average similarity of genre profiles to other countries. On the left side, countries with highest mainstreaminess; on the right side, countries with lowest mainstreaminess

Country	Avg. sim.	Country	Avg. sim.
NL	0.67677	EE	0.53230
UK	0.67371	BG	0.52833
BE	0.66060	ID	0.45704
CA	0.65586	GR	0.45162
ES	0.64279	HU	0.45156
PT	0.64021	RO	0.43108
FR	0.64000	JP	0.36894
AU	0.63917	CN	0.36652
NO	0.63090	IR	0.36369
RU	0.62583	SK	0.30447

#### Content-based recommendation

We implemented two content-based approaches, based on different data sources. We fetch for each artist (i) the mood descriptors from Allmusic and (ii) the links in the artist’s Wikipedia^19^ page.^20^ We assume that artists that share moods and links are more similar. Each artist is eventually represented by a set of moods and a set of Wikipedia links, based on which two content-based recommenders are constructed. To estimate similarity between two artists, we calculate the Jaccard index between their sets of moods and between their sets of links, i.e., we compute the share of overlapping elements in both artists’ item sets, separately for mood and for links. Artists similar to the ones listened to by the target user are then determined, weighted, aggregated, and ranked in a similar way than in the CF approach. Eventually, the *N* artists with highest scores, not known by the target user, are recommended. In our experiments, we considered up to $K=25$ most similar artists for each artist in the target listener’s training set.

#### Hybrid recommender

In order to create a hybrid recommender, we follow a late fusion strategy by integrating the recommendations of the content-based and the collaborative filtering algorithms. To this end, we first median-normalize the ranking scores given by the two recommenders to fuse. For artists suggested by both recommenders, we compute the new score as the arithmetic mean of both original scores; for all others, we take the original normalized scores. Based on the ranking obtained by sorting with respect to the new scores, we eventually recommend the top *N* artists.

#### Popularity-based recommendation

This recommender simply sorts all artists according to their overall playcounts and recommends the top *N*, excluding those which the target user already knows.

#### Random baselines

To contextualize the results of the recommender systems algorithms, we implemented two baselines: one that randomly selects *N* artists out of all artists the target user has not listened to, and one that randomly selects users and recommends *N* artists they listened to and are unknown to the target user.

### Experiments and results

**Fig. 8:**
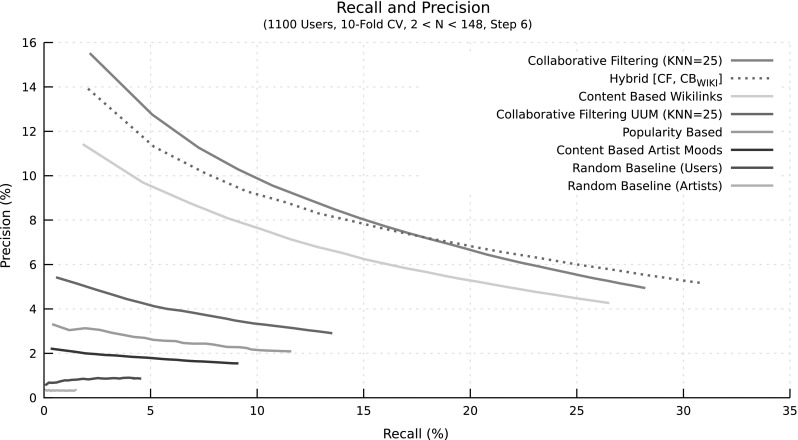
Precision/recall plot of various recommendation algorithms applied to a random subset of 1,100 users from the LFM-1b dataset. Tenfold cross-validation was used. Precision and recall are plotted for various numbers of recommended artists *N*, ranging from 2 to 148 using a step size of 6

For computational reasons, we ran the evaluation experiments on a subset of 1,100 users randomly sampled from LFM-1b. We performed 10-fold cross-validation on the listener level, i.e., we used $90\%$ of each target user’s listening history for training the system and the remaining $10\%$ as ground truth to evaluate the recommendations made by the system. We repeated this procedure ten times in a way that each listening event of the user occurs exactly once in the $10\%$ test data. Varying the number of recommended artists *N* allows us to investigate precision at different levels of recall. The results are shown in Fig. 8. As expected, CF and hybrid recommendations outperform all others. While CF has a slightly better performance when recommending a small number of artists *N* (higher precision at same recall level), the hybrid approach outperforms CF for larger numbers of recommendations (higher precision and higher recall). The content-based recommender based on Wikipedia links also performs considerably well, in contrast to the mood-based one, for which data seem too sparse. All other approaches perform substantially worse. Among the baselines, the random user selection performs slightly better than the random artist selection, which is due to the fact that the former tends to recommend artists that are more frequently listened to, while the latter performs a completely random selection.

## Conclusions and future work

In this article, we presented the LFM-1b dataset to support large-scale experimentation for tasks in music information retrieval and music recommender systems. The dataset can be downloaded from http://www.cp.jku.at/datasets/LFM-1b and provides information on the level of artists, albums, tracks, and users, as well as individual listening events. In addition to this content seen in other datasets as well, a unique feature of the LFM-1b dataset—next to its size—is the inclusion of detailed additional user-specific descriptors that model music preferences and consumption behavior. We analyzed the dataset’s properties and provided insights from an investigation of country-specific music taste, both in terms of genre preferences and their similarities between different countries. We strongly believe that the LFM-1b dataset, if not becoming a standard in benchmarking user-aware music recommendation approaches that go beyond rating prediction, will at least nicely complement existing datasets.

While the LFM-1b dataset can be used for experimentation in music retrieval and recommendation, particularly for collaborative filtering, demographic filtering, and personalized approaches, we contemplate several extensions. In particular, we would like to add audio-based features that allow to build music content-based recommenders and retrieval systems. While audio is generally not available for the tracks in the dataset, preview snippets provided by several online music stores could be acquired and audio features computed thereon. In addition to audio descriptors, features modeling the music context or background, such as TF$\cdot$IDF weights computed on web pages related to artists could be included. We are currently also gathering and preparing lyrics and text features computed thereon and plan to release them soon. These text-based features will enable tasks such as semantic querying by lyrics or artist characteristics. Finally, we are investigating additional user-specific features relating to music consumption behavior, which we plan to include in a possible extension of the current dataset.

## Footnotes

1
https://developer.spotify.com/web-api.

2
http://www.last.fm/api.

3
https://developers.soundcloud.com/docs/api/guide.

4
http://www.music-ir.org/mirex/wiki.

5
http://www.sigkdd.org/kdd2011/kddcup.shtml.

6
http://labrosa.ee.columbia.edu/millionsong.

7
http://www.kaggle.com/c/msdchallenge.

8
http://www.cp.jku.at/datasets/MMTD.

9
http://www.artofthemix.org.

10
http://mi.soi.city.ac.uk/blog/codeapps/the-magnatagatune-dataset.

11
http://www.last.fm/api/tos.

12
http://www.last.fm/api/show/tag.getTopTags.

13
http://www.last.fm/api/show/tag.getTopArtists.

14
http://www.last.fm/api/show/user.getRecentTracks.

15
http://www.last.fm/api/show/artist.getTopTags.

16
http://www.allmusic.com.

17
http://www.freebase.com.

18 Please note that these numbers are 20 and 100%, respectively, for the listed countries, when using the Allmusic genres for indexing.

19
http://en.wikipedia.org.

20 To determine the correct Allmusic and Wikipedia pages for a given artist, we implemented several heuristics and filtering pipelines.
